# Willingness and acceptability of cervical cancer screening among women living with HIV/AIDS in Addis Ababa, Ethiopia: a cross sectional study

**DOI:** 10.1186/s40661-015-0012-3

**Published:** 2015-09-18

**Authors:** Netsanet Belete, Yosief Tsige, Habtamu Mellie

**Affiliations:** Ethiopian Public Health Institute, Health System Research Directorate, Addis Ababa, Ethiopian; Addis Ababa University, Allied School of Health Science, Addis Ababa, Ethiopian; Debre Markos University, College of Medicine and Health Science, Department of Public Health, Debre Markos, Ethiopia

**Keywords:** Cervical cancer, HIV/AIDS, Screening, Willingness, Acceptability, Ethiopia

## Abstract

**Background:**

In Ethiopia, cervical cancer (CC) ranks the 2nd most frequent cancer and the country had 27.19 million women at risk of developing the disease though only 0.6 % women age 18-69 years was screened every 3 years. Nearly a quarter (22.1 %) of southern Ethiopia HIV (Human Immunodeficiency Virus) infected Women were positive for precancerous cervical cancer. Doing regular screening can prevent the disease by around half (45 %) of the cases in age of 30s and three quarter (75 %) cases in 50s and 60s.In the presence of high risk for acquiring cervical cancer among HIV patients, willingness and acceptance of the screening is low in Addis Ababa, Ethiopia thus the current study was aimed to assess willingness and acceptability of cervical cancer screening and its determinants among women living with HIV/AIDS in Addis Ababa, Ethiopia.

**Method:**

A facility based cross sectional study was conducted among HIV positive women attending HIV treatment centers in Addis Ababa. The respondents were identified using systematic random sampling method. Data was collected using pretested questionnaire and were entered in to Epi-info version 3.5.1 software and exported in to SPSS version 20 statistical package for analysis. The criterias for entering independent variables into multivariate analysis were having p-value 0.05 or less at bivariate analysis and not co-linear.

**Result:**

One third (34.2 %) of participants knew cervical cancer and two third (62.7 %) were willing for the test though only a quarter (24.8 %) were accepted the test. The independent variables significantly associated with acceptance of screening were educational level, source of information, awareness for the test and preventability of the disease.

**Conclusion:**

In current study willingness and acceptance of CC (cervical cancer) were low thus organizations working on cancer and HIV/AIDS should establish cervical cancer screening program and further enhance awareness creation.

## Background

In women, cervical cancer is the fourth most common cancer accounting about 20.4 % of all cancers globally, with an estimated 528,000 new cases in 2012 [1, 2]. Majority (85 %) of the global burden occurs in the less developed regions, where it accounts for almost 12 % of all female cancers. In sub-Saharan Africa, 34.8 new cases of cervical cancer are diagnosed per 100, 000 women annually and it happens in about 60 % women living with HIV infection [3–5].

There were an estimated 266,000 global deaths from cervical cancer in 2012, accounting for 7.5 % of all female cancer deaths. Almost nine out of ten (87 %) CC deaths occur in less developed regions. In sub-Saharan Africa, the mortality is 22.5 per 100,000 though it is less than 2 per 100,000 in Western Asia, Western Europe and Australia/New Zealand [1, 2].

In 2014, the expected diagnosis and death from invasive cervical cancer in America was 12,360 and 4,020 respectively [6].

More than one fifth of all new cervical cancer cases globally are diagnosed in India. In the country, the disease ranked 2nd cause of female cancer. According to 2012 estimate, the country annual diagnosed cases and deaths from the disease was 122,844 and 67,477 respectively [2, 7]. In Taiwan the incidence rate of the disease among HIV infected women was 712.08/100,000 person-years [8].

In 2012, Ethiopia has a population of 27.19 million women aged 15 years and older who are at risk of developing cervical cancer. Current estimates indicate that every year 7095 women are diagnosed with cervical cancer and 4732 die from the disease. The disease ranks 2nd most frequent cancer among women. The disease has crude incidence rate of 16.3 per 100,000 populations per year in the country. Only 0.6 % women age 18–69 years was screened every 3 years [9].

As evidences showed HIV infected women are at risk for CC [10–13]. Cervical cancer is about 7.9 times more common in HIV infected women than none infected ones [10]. Cervical squamous intraepithelial lesion was about 7.04 times higher in HIV positive compared HIV negative women [12]. Nearly a quarter (22.1 %) of Southern Ethiopia HIV-Infected Women were positive for precancerous cervical cancer [13].

Cervical cancer which is caused by persistent infection with human papillomavirus [1, 7, 12, 14–17] is preventable disease, yet the number of cases globally is expected to almost double by the year 2025 [18]. Doing regular screening (no more than once every three to five years) can prevent the disease by around 45 of the cases in age of 30s and 75 % cases in 50s and 60s [19, 20]. Screening can be done using Pap smear [20], human papillomavirus testing [21] and visual inspection with acetic acid [22].

In the presence of high risk for acquiring cervical cancer among HIV patients, awareness and acceptance of the screening is low. In Boston, of eligible women for screening about 53.0 % had not undergone screening [23]. In Kenya teaching and referral hospital the self-reported screening uptake was 17.5 % [24]. About half (56.2 %) of HIV positive Nigerian women were aware of cervical cancer. In same country, only one every ten (9.4 %) of HIV positive women were screened for cervical cancer [25].

In Addis Ababa, Ethiopian women had very low awareness of cervical cancer and the etiology of cervical cancer was thought to be due to breaching social taboos or undertaking unacceptable behaviors. As a result, the perceived benefits of modern treatment were very low [26].

Although cervical cancer is a leading cause of cancer related morbidity and mortality among women in Ethiopia, its screening coverage as part of HIV care was low, only covers less than 1 % due to have no national screening program [18, 27, 28]. Hence the current study used to assess the willingness and acceptability of cervical cancer screening and its determinants among HIV positive women. The obtained information will be used for decision makers and organizations working on cancer and HIV/AIDS to consider and integrate cervical cancer screening as part of HIV/AIDS diagnosis and treatment guideline since there is no prior local evidence up to our knowledge.

## Methods and materials

### Study design and setting

A cross-sectional study design using both quantitative and qualitative research method was conducted on HIV treatment centers in Addis Ababa which is the capital city of Ethiopia. In the town, there are 45 hospitals, 72 health centers, and 43 health posts owned by ministry of health [29, 30]. These service sites were providing HIV diagnosis and treatment and its related supportive services. There are about 210,306 people living with HIV/AIDS in Addis Ababa of which 124,609 are women [31].

All HIV infected women above age of 17 years and coming for chronic HIV care in public health institutions of Addis Ababa during study period were the study populations.

### Sample size determination and sampling strategy

The sample size for quantitative part was determined by a single population proportion formula using 95 % confidence interval (a = 0.05), 5 % margin of error (d) and the proportion (p) of cervical cancer screening in women living with HIV/AIDS from prior studies, which was 27.0 % [32].

$$ n = {\left({Z}_{a/2}\right)}^2\left(p\left(1-p\right)\right)/{d}^2 $$

The final sample size after adding 10 % contingency was 333.

For qualitative part two women from each referral hospital and one woman from each health centers was selected for in-depth interview.

Among Addis Ababa regional health bureau owned public health institutions, three referral hospitals and eight health centers providing ART service were selected for study by lottery method. To calculate the sampling interval for quantitative study, the HIV patient flow for the past six consecutive months before data collection period was taken from each selected health institutions and added for approximation of future sampling frame. Selection of participants was done using systematic random sampling method based on arrival order of HIV infected women to the health institution for medical care. The staring participant was selected by lottery method from sample interval. The interviewed participant’s corresponding medical record was reviewed for assessing laboratory measurements like CD4 count and clinical stages of HIV/AIDS.

For qualitative data, purposive sampling method was used to select women for the in-depth interview in selected health institutions. Women who were not selected for interview in quantitative study were included for qualitative part.

### Data collection procedure and data quality management

For quantitative study, a structured questionnaire which was developed by reviewing different literatures was used for data collection. The questionnaire was translated in to local language (Amharic) and back translated to check consistency. The data collection tool was pretested among 16 patients and identified errors were corrected accordingly. The outcome variable of the study was cervical cancer screening acceptability. For the qualitative aspect a semi-structured interview guide was prepared in English and translated to local language of Amharic version. The in-depth interview was tape recorded and note was taken during interview.

To maintain quality of data; standardized and pre- tasted data collection tools were used for both quantitative and qualitative studies. Appropriate training was given for data collectors and supervisors. Daily supervision was carried out by supervisors and principal investigators to check completeness of the questionnaire. The quantitative data was entered twice by trained data clerk to check correct data entry. In addition at the end of data entry data cleaning were done using frequencies, cross tabulations, sorting and listing to check missed values and outliers. Errors identified during data collection were corrected accordingly at the field and those errors occurred during/after data entry were corrected by revising the original questionnaire.

### Operational definitions

- Willingness to be screened for cervical cancer - if a women willing for testing but not decided to be screened in the near future [25].
- Acceptance to be screened for cervical cancer- if a women willing for testing and decided to be screened in the near future” [25].
- Knowledgeable about cervical cancer- if participant answers 7 questions out of 13 Knowledge assessment questions.

### Data analysis

For quantitative data, code was given and entered in to Epi-info version 3.5.1 software and exported in to SPSS version 20 statistical package for analysis. Multi-co-linearity was checked using Pearson correlation, tolerance or variance inflation factor and there were not multi-co-linearity. Independent Variables associated with outcome variable with p-value 0.05 or less at bivariate analysis and not co-linear were entered into multivariate analysis via binomial logistic regression to get adjusted predictors. Descriptive statics of continuous variables were presented using mean, median and discrete variables were presented using percentage, and tables. *P*-value <0.05 was used as cut off point of statistical significance for analytical analysis.

The qualitative in-depth interview was translated to English version by arranging the points according to forwarded questions. Then framework analysis method was employed to grasp the detail information.

### Ethical issue

The study proposal was approved by the institutional review board of Addis Ababa University. Letter from the Research Ethics Committee was submitted to Addis Ababa regional health bureau to get permission for conducting the study. To protect anonymity and confidentiality of the participants, both data collectors and supervisors were working in ART clinics in addition personal identifier data was not collected. Informed written consent was obtained from respondents after explaining the purpose of the study.

## Results

A total of 322 study participants were included with a response rate of 96.7 % and about 14 in depth interviews were conducted based on saturation of information.

### Characteristics of the study participants

The dominant ethnicity and religion were Amhara (53.7 %) and orthodox Christianity (71.7 %) respectively. Mean age of the study participants was 35.65 (SD ±10.17). Most of them were educated (78.3 %) and about half of them were have no regular source of income (51.2 %), had experience of pregnancy 1–2 times (47.5 %), diagnosed as having HIV before 6–10 Years ago (49.1 %). Most of (91.9 %) the participants were initiated ART and were WHO clinical stage 2 (34 %) HIV patients.

On card review, CD4 count of 211 (65.5 %) participants were less than or equal to 500 cells/ul, and the remaining 111(34.5 %) were CD4 count greater than 500 cells/ul. Most of the participants in the qualitative were attended secondary education, married and were between the age of 29 and 35.

### Cervical cancer awareness, knowledge and test acceptance

Of the whole participants 110 (34.2 %) know about cervical cancer. And their major sources were health professionals during contact on routine HIV chronic care 59 (53.6 %), media 64 (58.2 %), reading books 13 (11.8 %), and friends 11(10.0 %) and the least was from family 3 (2.7 %). Regarding knowledge assessment only 81 (25.1 %) was found to be knowledgeable.

Even though they didn’t have detail knowledge regarding the disease, most of the in-depth participants were aware of cervical cancer. Some of the participants agree as it is the cancer of the female reproductive tract. Medias are mentioned as a source of knowledge about the disease for most of the participants and some of them also experience the disease in their family members and neighbors.

Majority of participants 97 (88.2 %) believe as the disease is a preventable. Similarly from the qualitative part, most of the participants believed as cervical cancer is a preventable disease. A young respondent mention that *“one can get prevented from cervical cancer by having the screening test regularly”.*

About one third 31.4 % (101) participants knowing the availability of the screening procedures for the disease and about one third (62.7 %) were willing to screened for cervical cancer and a quarter (24.8 %) of them were decided to screened in the near future.

Most of the participants repudiate to take the screening test due to assuming, the test is time consuming 43 (35.8 %) (Table 1). The mentioned reasons in in depth interview were high cost of the test and timing consuming, fear of result of the test (being diagnosed as having cervical cancer), and a recently HIV diagnosed woman noted that *“the word you have a cancer diagnosis is really irritating beside my HIV, I think I will get hopeless, if I am diagnosed as having cervical cancer”.*

**Table 1:** Reason for not having screening test among study participants in Addis Ababa, 2014

Reason for not having the screening test	Variable	Frequency	Percentage (%)
	High cost of the test	36	30.0
	Religious denial	12	10.0
	Partner acceptance	12	10.0
	Time consuming	43	35.8
	Recently delivered/Pregnant	16	13.3
	Fear of test result	37	30.8
	Lack of female screeners	16	13.3
	No reason	25	20.8
	Others	9	10.8

Only 37 (11.5 %) of the study participants were ever tested for cervical cancer in their life time. The time of screening was before HIV/AIDS diagnosis (29.7 %), within one year of HIV/AIDS diagnosis (32.5 %) and after one year of HIV/AIDS diagnosis (37.8 %). Of those who get the screening test, about 11(29.7 %) were positive for the test.

In multiple logistic regression, after adjustment for potential cofounders, the factors that enhance accepting of screening were being in age group of 40–49, 50–59 and >60 years compared <29; having above 12 grade educational level compared to read and write; having regular source of income; getting information about cervical cancer from health professionals, having awareness of the test and needing the screening to take early measure. Respondents not knowing cervical cancer as preventable were less likely to accept the screening (Table 2).

**Table 2:** The association of variables with accepting of cervical cancer screening among study participants, 2014

Variable	(AOR, 95 % CI)
Socio-demographic characteristics	
Age in completed years	
<29 Years	1
30-39 Years	0.96 (0.372, 1.671)
40-49 Years	3.8 (1.212, 12.290)*
50-59 Years	4.2 (1.505, 17.482)*
>60 Years	8.2 (1.104, 61.543)*
Level of education	
Read and write	1
Primary Education (1–8)	1.2 (0.417, 3.846)
secondary Education (9–12)	1.6 (0.479, 5.600)
Higher education above 12	1.2 (1.313, 5.269)*
Regular source of income	
Yes	3.2 (1.346, 8.005)*
No	1
First most cause of cervical cancer	
Viral	1.8 (0.011, 3.895)
I don't know	0.619 (0.001, 2.233)
Others	1
Health professional source	
Yes	6.0 (1.440, 83.933)*
No	1
Is cervical cancer preventable	
Yes	1
No	0.07 (0.001, 0.249)*
Aware of the test used	
Yes	3.6 (1.395, 33.76)*
No	1
Reason of testing to take early measure	
Yes	9.9 (1.423, 309.316)*
No	1
High cost of the test	
Yes	1.001 (0.811, 29.268)
No	1
WHO Clinical staging	
WHO stage 1	1
WHO stage 2	0.68 (0.079, 5.870)
WHO stage 3	0.67 (0.042, 10.675)
WHO stage 4	0.011 (0.463, 3.282)

*AOR* adjusted odds ratio, *WHO* world health organization

* = statistically significant

## Discussion

In this study one third (34.2 %) participants knew the availability of screening test for CC and this finding was similar with HIV positive women in Lagos, Nigeria (34.5 %) [25] and it was higher than two studies in Nigeria (15.5 %) [33], (6.5 %.) [34]. The possible reasons for discrepancy of the result might be variation in study populations i.e. all women [33, 34] vs HIV infected women in current study, regional state or country specific promotion policy variations, variations in involvement of the CC education in media and its exposure and differences in socio-cultural condition.

In current study majority of participants mentioned as health professionals were their main source of information for CC and the finding is in agreement with prior studies [33, 35–37].

The current finding of two third participants (62.7 %) willing to be screened for cervical cancer were lower than a study done in Nigeria (96.5 %) [38] and Mozambique (84 %) [39] and the difference might be attributed by variations in health policy on promotion of CC, variations on awareness creation using mass-media and socio-economic variations.

The current study revealed as one every ten (11.5 %) participants were ever tested for CC in their life time and this is in line with a study in rural Mozambique (11 %) [39], Sokoto, Nigeria, (10 %) [40], two studies in Kenya (12.3 %) [41] and (17.5 %) [25] and HIV positive women in Lagos, Nigerian (9.4 %) [25]. The finding was higher than two studies in Ogun State, Nigeria (4.8 %) [34] and (9.5) [42] and it was lower than a study among HIV-positive women Ottawa, Ont (58 %) [23]. The reason of being higher than Ogun State, Nigeria [34, 42] might be attributed by variations of study populations i.e. all women in [35] since HIV infected women may frequently visit health institutions and can get health professionals which are the main source of information for screening [33, 35–37].

The current finding of most of the participants not willing to take the test due to assuming that the test is time consuming (35.8 %), fear of being positive for the test (30.8 %) and high cost of the test (30.0 %) was in agreement with the a similar study in HIV positive women in Lagos, Nigerian [25] and Kenya [41].

In this study, participants having above 12^th^ grade educational level compared to read and write, and having awareness about CC were 1.2 and 3.6 times respectively more likely accepting the screening and the finding was in parallel with a study among HIV positive women in Lagos, Nigerian in which having a tertiary education were enhancing screening by 1.4 times (OR = 1.4; 95 % CI: 1.03-1.84) and those aware of cervical cancer were 1.5 times (OR: 1.5; 95 % CI: 1.2-2.0) more likely accepting the screening [25].

In current study, participants who were getting information about cervical cancer from health professionals were 6 times more likely accepting the screening and this finding was supported by prior studies [33, 40].

## Conclusion and recommendation

One third (34.2 %) participants knew cervical cancer and two third (62.7 %) were willing for the test though only a quarter (24.8 %) were accepted the test. After adjustment in multivariate analysis, the factors that enhance accepting of screening were being in age group of 40–49, 50–59 and >60 years compared <29; having above 12^th^ grade educational level compared to read and write; having regular source of income; getting information about cervical cancer from health professionals, having awareness of the test, needing screening to take early measure and knowing cervical cancer as preventable disease. Thus organizations working on cancer and HIV/AIDS should further work to enhance awareness, and acceptability of the test. Ethiopian ministry of health should establish CC screening program since two third participants were willing for screening. Screening refusals as a result of assuming time consuming of the test, fear of the test result and anticipated high cost need to be addressed through advocacy and public mobilization. A further study with a large sample size is recommended to validate the current finding.

## Abbreviations

AIDS: Acquired immunodeficiency syndrome
ART: Antiretroviral treatment
CC: Cervical cancer
HIV: Human Immunodeficiency Virus
